# Obesity and Reproduction

**DOI:** 10.5935/1518-0557.20160037

**Published:** 2016

**Authors:** Joao Batista A Oliveira

**Affiliations:** 1 Center for Human Reproduction Prof. Franco Junior, Ribeirão Preto/SP - Brazil; 2 Paulista Center for Diagnosis Research and Training, Ribeirao Preto/SP - Brazil

**Keywords:** Obesity, reproduction, infertility, body weight

Obesity is associated with multiple interrelated disorders such as insulin resistance/diabetes, hypertension, dyslipidemia, sleep apnea - all contributing collectively to the diagnosis of a metabolic syndrome, that reduces life expectancy (Flegal *et al*., 2013). Metabolic changes in obesity may also affect reproduction.

In women, obesity is associated with a higher incidence of ovulatory disorders and idiopathic infertility (ASRM, 2015). On the other hand, obese women under treatment for infertility may face additional problems, such as the need for higher doses of drugs to induce/stimulate ovulation, oocyte morphological changes, reduction in fertilization and implantation rates, and embryo quality (ASRM, 2015; Provost *et al*., 2016). Compared to women of normal body weight, obese women submitted to IVF may present reduced rates of clinical pregnancy and live births, with an increased rate of abortion (Provost *et al*., 2016). In addition, obese pregnant women have a higher incidence of maternal and fetal complications, such as gestational diabetes, hypertensive disorders of pregnancy and increased perinatal morbidity/mortality (Aune *et al*., 2014).

With respect to men, male obesity has been linked to reduced rates of pregnancy and live births (Campbell *et al*., 2015). However, studies on specific relationships between semen parameters and obesity have been contradictory. Although different studies have shown correlations between increased obesity and changes in sperm parameters, although selectively (MacDonald *et al*., 2010, Sermondade *et al*., 2013, Campbell *et al*., 2015), others did not report adverse effects (Bandel *et al*., 2015). On the other hand, recent studies point to a negative association between body weight and the very integrity of sperm DNA (Fariello *et al*., 2012; Taha *et al*., 2016); however, these results are not unanimous (Bandel *et al.*, 2015; Campbell *et al.*, 2015). Spermatogenesis requires a controlled testicular environment and intact endocrine signaling through the hypothalamic-pituitary-testicle axis; and the impact of obesity on fertility can be attributed mainly to the endocrine mechanisms that alter this relationship (MacDonald *et al*., 2010; Fariello *et al*. 2012; ASRM, 2015). Moreover, the preferential buildup of toxic substances and fat-soluble endocrine disruptors in adipose tissue, and hyperthermia resulting from the buildup of adipose tissue around the scrotum cause oxidative stress to the testes, thus broadening these alterations (Fariello *et al.*, 2012; Sermondade *et al*., 2013, Taha *et al.*, 2016).

In conclusion, even considering the controversies, weighty reduction and lifestyle interventions should be included in the recommendations to obese infertile couples.
